# Thyrotoxic periodic paralysis in an adolescent male: A case report and literature review

**DOI:** 10.1002/ccr3.3558

**Published:** 2020-11-23

**Authors:** Luke He, Veronica Lawrence, Wayne V. Moore, Yun Yan

**Affiliations:** ^1^ Department of Emergency Medicine Creighton University Phoenix AZ USA; ^2^ School of Medicine University of Missouri‐Kansas City Kansas City MO USA; ^3^ Department of Emergency Children's Mercy Kansas City MO USA; ^4^ Department of Endocrinology Children's Mercy Kansas City MO USA

**Keywords:** Adolescent, Grave's disease, hypokalemia, periodic paralysis, thyrotoxicosis

## Abstract

Thyrotoxic periodic paralysis (TPP) is rarely seen in children and adolescents. Clinical manifestations in children and adolescents may vary. It is important for clinicians to be aware of this rare and life‐threatening condition.

## Graphical abstract

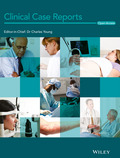

## 1 INTRODUCTION

Thyrotoxic periodic paralysis (TPP) is an uncommon hyperthyroidism‐related condition characterized by abrupt onset muscle weakness and hypokalemia resulting from rapid intracellular shift of potassium. TPP is primarily reported in adult males in Asian populations, including Chinese, Japanese, Vietnamese, Filipino, and Korean.^1^, ^2^ Its incidence in non‐Asian populations such as Caucasians, African Americans, and Hispanics is very low. PTT is very rarely seen in the pediatric population.^3^ A number of TPP cases in adults have been reported in Western countries recently. TPP is commonly misdiagnosed in Western countries because of its similarities to familial periodic paralysis.^4^ We report a case of an Asian adolescent male presenting with acute‐onset paralysis and severe hypokalemia and highlight the importance of recognizing TPP.

## 2 CASE REPORT

A 17‐year‐old Vietnamese American male presented to the emergency department after an episode of syncope, muscle weakness, and difficulty breathing. Two months prior, he began having episodes of tachycardia and was subsequently evaluated by Cardiology. He was diagnosed with Graves' disease with a TSH of 0.007 mIU/mL, free T4 > 7 ng/dL (0.8‐1.9), and positive thyroid‐stimulating immunoglobulin and was started on atenolol. He was then seen by Endocrinology and started on methimazole 10 mg, 3 times daily. Thyroid ultrasonography revealed markedly increased internal vascularity within the thyroid gland. On the next morning, he reported feeling weak. His father found him on the floor of his bedroom, weak and unable to move, approximately 30 minutes after his father “heard a thud upstairs.” He recalled that his legs “gave out” and he “hit his face on a table”. In the emergency department on presentation, he was tachycardic at 116 bpm, blood pressure 131/50 mm Hg with respiratory rate 24 BR/min. He had diffused and significant muscle weakness in all extremities, including grip strength. He was alert and awake without complaint of headache, and physical exam revealed no papilledema during the emergency department and PICU stay; therefore, no brain imaging was obtained.

Basic metabolic panel revealed potassium 1.6 mmol/L (3.5‐5.2) and magnesium 1.6 mmol/L (1.6‐2.3). The rest of his metabolic panel was unremarkable. He had EKG changes consistent with hypokalemia with U waves. The EKG also revealed an atrial rhythm with first degree AV block, intraventricular conduction delay, and QTc prolongation at 588 (<450). His chest x‐ray was normal. Normal saline was administered, and potassium replacement was given with 40 mEq of KCl followed by D5 NS with 40 mEq/L KCl at the maintenance rate (90 mL/hr). He was also given one dose of magnesium intravenously. His potassium level returned to normal range at 4.6 mmol/L and muscle strength returned completely after 24 hours of treatment. Four months after he was discharged home, he underwent radioactive iodine ablation, which he tolerated well. He is now 2 years status post radioactive iodine ablation and has not had a recurrent paralysis episode. He takes thyroid hormone replacement daily and is doing well.

## 3 DISCUSSION

Because TPP is not often seen in the United States and other Western countries, it can be easily missed. Hypokalemia, in particular, has broad differential diagnoses. Our patient was an Asian American recently diagnosed with Graves' disease, who presented with tachycardia and significant weakness in all extremities. His laboratory evaluation revealed hypokalemia. His EKG revealed changes consistent with hypokalemia including U waves.

The age of onset of TPP symptoms is typically between 20 and 39 years. TPP is very rare in children and adolescents, making the diagnosis in this age group challenging. In the past 2 decades, fewer than 20 cases of TPP in adolescents have been reported.^5^, ^6^, ^7^, ^8^, ^9^ Ethnicities represented include Chinese,^10^, ^11^ Hispanic,^12^ Korean,^6^, ^8^, ^13^, ^14^ and Vietnamese.^15^ One case was recently reported in a 14‐year‐old African American boy.^7^ Among previously reported cases of pediatric TPP (Table 1), only one patient was female. All 15 patients had Graves' disease and hypokalemia, and potassium was usually less than 3.0 mmol/L (potassium was 1.6 mmol/L in our case). The degree of initial hypokalemia has a direct correlation with the severity of paralysis. Our patient presented with shortness of breath and tachycardia. Fatal ventricular arrhythmia^16^ and respiratory failure^17^ have been reported. To avoid such fatal complications, early recognition and prompt treatment is essential. In the review of previously reported cases, the 15 adolescents recovered well from TPP and only two cases had a recurrent episode after discharge (Table 1).

**Table 1:** Summary of age, sex, ethnicity, laboratory evaluation, treatment, and follow‐up

#	Age	Ethnic	Sex	K^+^ (mmol)	T3 (ng/dL)	FT4 (ng/dL)	TT4 (µg/dL)	TSH (mU/L)	Therapy	Recurrent paralysis after discharge	Reference
1	16	Chinese	M	2.0	404	NA	16.5	<0.03	Propranolol, methimazole, RIA	0.5 yr follow‐up: none	Miller et al^10^
2	14	Chinese	M	2.2	840	1.98	NA	<0.03	Propranolol, carbimazole	NA	Wong et al^11^
3	15	Vietnamese	M	1.8	NA	4.66	NA	0.001	Carbimazole, thyroxine	NA	Schalin‐Jantti et al^15^
4	14	Korean	F	2.3	183	3.8	NA	0.01	β‐adrenergic blocker, antithyroid drug	3 yr follow‐up: none	Oh et al^8^
5	16	Korean	M	2.5	180	1.87	NA	0.09	Methimazole	2 recurrent episodes	Ahn et al^13^
6	17	Korean	M	2.2	285	3.43	NA	<0.01	Propranolol, methimazole	1 recurrent episode	Cho et al^14^
7	13	Asia	M	2	253	5.72	NA	NA	Propranolol, methimazole	NA	Jones et al^5^
8	16	Korean	M	2.7	295	2.1	NA	<0.025	Propranolol, methimazole	1 yr follow‐up: none	Jung et al^6^
9	17	Hispanic	M	<3.5	>800	NA	22.4	<0.01	β‐adrenergic blocker, methimazole	1 yr follow‐up: none	Al‐Zubeidi et al^12^
10	15	Hispanic	M	1.5	608	NA	23.8	0.04	Methimazole	3 yr follow‐up: none	Al‐Zubeidi et al^12^
11	15	Chinese	M	1.6	240	2.8	12.6	0.008	Methimazole, atenolol	NA	Thornton et al^25^
12	16	Korean	M	2.5	246	2.46	NA	<0.008	Methimazole	2 yr follow‐up: none	Roh et al^9^
13	14	Korean	M	3.1	615	4.97	NA	0.06	Propranolol, propylthiouracil	3 yr follow‐up: none	Roh et al^9^
14	14	African American	M	2	>651	>7	16.8	<0.005	Propranolol, methimazole	0.5 yr follow‐up: none	Glass et al^7^
15	17	Vietnamese	M	1.6	NA	>7	NA	0.007	Methimazole, atenolol, RIA	2 yr follow‐up: none	In this report

Abbreviations: FT4, Free T4; NA, not available; RIA, radioiodine ablation; T3, triiodothyronine; T4, thyroxine; TSH, thyroid‐stimulating hormone; TT4, total T4.

The pathogenesis of TPP is not entirely clear. Recent studies have shown that TPP is a channelopathy related to mutations in the KCNJ18 gene encoding Kir2.6, a skeletal muscle‐specific Kir channel protein. Mutant Kir2.6 proteins significantly reduce cell membrane abundance and could interfere with the excitability of skeletal muscle cells.^18^, ^19^ TPP is more commonly reported in Asian males, with onset usually in the third decade of life.^20^, ^21^ More than 95% of TPP is found in men, despite most thyrotoxicosis being found in women.^22^ TPP patients may present with hypokalemia, as did our patient, due to the intracellular shift of potassium throughout the body when the Na+/K+–ATPase activity in the cell membrane goes awry and is hyperactivated, as it does when influenced by beta‐adrenergic catecholamines or insulin, which may explain why hypokalemia presents in patients in a hyperadrenergic state secondary to hyperthyroidism.^23^ TPP episodes usually occur during rest after exercise or after a high‐carbohydrate meal.^24^

Hypophosphatemia and hypomagnesemia may also be present in patients with TPP, contributing to weakness. Our patient had normal phosphorus and low normal magnesium. In TPP the total body potassium is unchanged, unlike other conditions such as decreased potassium intake or losses of potassium through sweat, urine, or gastrointestinal tract. The differential should also include neurological etiologies anywhere, including the brain, the spinal cord, the peripheral nerves, and the neuromuscular junction. The cause of both the weakness and cardiac conduction abnormalities seen in TPP is hypokalemia. Patients with TPP may experience a spectrum of symptoms including muscle aches, soreness, weakness, and flaccid paralysis. The attacks generally begin at the lower extremities and then move to girdle muscles and upper limbs, without sensory deficits.^24^, ^25^ Deep tendon reflexes are diminished or absent,^20^ as seen in our patient's initial presentation. Acute neuromuscular paralysis related to hypokalemia is an important clinical entity with diverse etiology, but the treatment and prognosis differ depending on the etiology. A differential may include familial hypokalemic periodic paralysis (FHPP), which typically occurs earlier in life and is more likely to have a family history. Electrocardiogram changes may include sinus tachycardia due to the hyperadrenergic state, prolonged QT‐U interval due to hypokalemia, and prolonged PR due to thyrotoxicosis.

Because TPP is not often seen in Western countries, the diagnosis can easily be missed. . Our patient was an Asian American and recently diagnosed with Graves' disease. He presented with tachycardia and notable muscle weakness in all extremities. His laboratory evaluation revealed hypokalemia and hypomagnesemia, and his EKG revealed changes consistent with hypokalemia and U waves.

Stabilization of patients with TPP should emphasize cardiac abnormalities and normalization of serum potassium. Potassium replacement is necessary immediately. After initial administration of potassium, most patients will have an increase in potassium, while up to 25% of patients may have an initial drop with stabilization over a few hours. Rebound hyperkalemia can occur during potassium supplementation. Excessive doses of potassium can lead to hyperkalemia once potassium shifts to the extracellular space. If potassium supplement is not enough, some reports suggest using IV or oral propranolol for reversing the hypokalemia via blocking the beta‐adrenergic receptor. This treatment may also prevent attacks before an euthyroid state is reached.^25^

## 4 CONCLUSION

Thyrotoxic periodic paralysis is a rare cause of acute paralysis and can lead to cardiac arrhythmias and death without accurate diagnosis and appropriate treatment. Since TPP is rare in Western countries, especially among pediatric patients, many physicians may be unfamiliar with this condition. Efficient evaluation and timely treatment is crucial to prevent complications, especially fatal arrhythmias. Acute paralysis with hypokalemia should also prompt physicians to evaluate thyroid function as a differential diagnosis.

## CONFLICT OF INTEREST

None declared.

## AUTHOR CONTRIBUTIONS

YY and VL: managed the patient. LH: led data collection, conducted literature search, and wrote the initial draft of the manuscript. LH, VL, WVM and YY: edited the draft into this manuscript. All authors approved the final version of the manuscript and agree to be accountable for all aspects of the work ensuring that questions related to the accuracy or integrity of any part of the work are appropriately investigated and resolved.

## ETHICAL APPROVAL

Written consent was obtained from the patient's parents.

## Data Availability

All data generated or analyzed during this study are included in this published article or in the data repositories listed in references.
